# Exploring the Nexus: Climate Change, Poverty, and Mental Health

**DOI:** 10.7759/cureus.97371

**Published:** 2025-11-20

**Authors:** Manpreet Singh, Sampriti Paul, Saptadweep Saha, Jutika Ojah, Bidyut Kr Das

**Affiliations:** 1 Community Medicine, Maulana Azad Medical College, New Delhi, IND; 2 Community Medicine, Gauhati Medical College and Hospital, Guwahati, IND; 3 Internal Medicine, Gauhati Medical College and Hospital, Guwahati, IND

**Keywords:** adaptation strategies, climate change, eco-anxiety, health equity, intersectionality, mental health, poverty, resilience, sustainable development, vulnerability

## Abstract

Climate change, poverty, and mental health are interconnected drivers of vulnerability that reinforce one another through complex social and environmental pathways. This narrative review synthesises evidence from PubMed, Scopus, Web of Science, and Google Scholar and comprises relevant research published in English between 1990 and 2025. Many people worldwide who are exposed to dangerous air pollution live in low- and middle-income nations, where economic hardships worsen climate-related challenges. Climate change exacerbates global economic difficulties and heightens poverty by increasing disease prevalence, causing forced migration, and destroying livelihoods. Additionally, communities repeatedly hit by climate disasters experience growing rates of depression, anxiety, and post-traumatic stress, with the most vulnerable groups suffering the most severe psychological effects. New findings from South and Southeast Asia also indicate that rising temperatures are increasingly linked to negative mental health effects, highlighting climate stress as an escalating public mental health issue. However, data remain fragmented, with few longitudinal or intersectional studies and limited economic assessments from low- and middle-income countries. The review underscores the need for integrated, equity-centred climate and mental health policies, cross-sectoral collaboration, and stronger health system resilience to mitigate these cascading risks.

## Introduction and background

Climate change refers to long-term alterations in weather patterns and average temperatures over extended periods. Although natural elements such as volcanic eruptions and solar cycles play a role, human activities have been the primary driver since the mid-20th century. This is largely due to the combustion of fossil fuels, deforestation, and industrial activities, which elevate the levels of greenhouse gases, such as carbon dioxide, methane, and nitrous oxide, in the atmosphere. These gases trap heat, enhancing the natural greenhouse effect and leading to further warming of the Earth's surface [1-6]. The impacts of climate change are evident in the form of increasing global temperatures (global warming), more frequent and intense extreme weather events (such as heatwaves, droughts, floods, and storms), melting glaciers, rising sea levels, ocean acidification, and changes in precipitation patterns. These phenomena pose risks to ecosystems, biodiversity, agriculture, water resources, human health, and infrastructure [7-14]. The major effects of climate change and their implications are summarized in Table 1.

**Table 1:** Summary of major climate change effects and their impacts.

Effect	Description
Global warming	Increase in average surface temperature [1,3,5,7,9]
Extreme weather events	More frequent/intense heatwaves, storms, floods [1,3,6,7,9]
Sea level rise	Melting ice and thermal expansion of oceans [1,3,5,9,15]
Biodiversity loss	Habitat shifts, species extinction [4,6,9,10]
Human health risks	Disease spread, heat stress, food insecurity [4,6,9,11,12]

Addressing climate change is a complex global issue that necessitates joint efforts from healthcare professionals, scientific experts, public health authorities, and policymakers to reduce its impact on health [16]. Understanding the role of microorganisms in climate change processes and their responses to environmental shifts is crucial for developing comprehensive climate models and mitigation strategies [17,18].

The economic repercussions of climate change are both significant and complex. By 2060, the global annual gross domestic product (GDP) is expected to decline by 1.0-3.3%, with the damage rate increasing at twice the pace of global economic growth [19]. The most significant negative economic consequences are expected to arise from the impacts on labor productivity and agriculture. However, the largest known economic impact is on agriculture, particularly in developing countries, owing to the sector's size and sensitivity to climate variations [20]. Warming is expected to cause substantial damage to agriculture in these countries over the next century, even with adaptation efforts. Developing countries, particularly in Africa and Asia, are projected to face the largest net economic consequences, whereas some higher-latitude countries may experience benefits in sectors such as tourism, energy, and health [21]. These economic challenges have profound implications for societal well-being, directly influencing poverty and mental health outcomes.

Climate change, poverty, and mental health are intricately linked, with each reinforcing the other. Those living in poverty are disproportionately affected by climate change because of their limited resources for adapting to environmental shifts. This vulnerability can lead to stress, anxiety, and other mental health challenges among adolescents. Financial hardship often restricts access to mental healthcare, exacerbating existing issues. Poor mental health can impede individuals from maintaining employment or education, potentially perpetuating their poverty. Climate change impacts mental health through natural disasters, displacement, and economic instability, leading to anxiety, depression, and posttraumatic stress disorder. Addressing these issues requires a holistic approach that acknowledges their interconnectivity. Green job initiatives can tackle poverty and climate change while enhancing mental health by promoting economic stability. Education plays a crucial role in helping lift people out of poverty, increasing climate awareness, and providing mental health management tools. Community-based solutions and strong social networks can offer mental health support, facilitate climate action, and create economic opportunities for women. Policy interventions are necessary for systemic change, including environmental regulations, access to mental health services, and poverty reduction strategies. Innovation and technology can contribute solutions through telemedicine, clean energy, and digital financial services. Addressing these interconnected issues requires cross-sector collaboration to develop effective solutions that create positive changes across all areas. Climate change acts as a risk enhancer, disrupting the conditions necessary for maintaining good mental health, including socioeconomic, cultural, and environmental factors [6].

Vulnerable populations are disproportionately affected by climate change owing to a mix of socioeconomic, environmental, and health-related factors. These groups include low-income individuals, minorities, and socially marginalized communities, who frequently lack the necessary resources and infrastructure to effectively respond to and recover from climate-related incidents [22]. The relationship between poverty and mental health is well-established, with poverty being a significant driver of mental illness [23]. Climate change-induced phenomena, including severe weather, food shortages, and displacement, can result in a range of mental health problems such as post-traumatic stress disorder, anxiety, depression, and substance use disorders [24].

Interestingly, the concept of "eco-anxiety" has emerged, reflecting the psychological distress resulting from awareness of the impacts of climate change. This phenomenon affects family planning decisions and disrupts cultural connections to nature, particularly among women and Indigenous communities [25]. Moreover, merely being aware of the climate crisis on an abstract level can evoke strong negative emotions, potentially leading to mental health problems, even among those not directly experiencing the tangible effects of climate change [24]. In summary, tackling the intricate relationship between climate change, poverty, and mental health requires a comprehensive strategy. This strategy should encompass support for climate change mitigation and adaptation efforts, address the root causes of collective violence and mental health disorders, and advocate for social and economic equity [22,23]. By integrating intersectional approaches into public policy and research, it is possible to identify and assist high-risk populations, thereby reducing mental health inequalities and enhancing overall well-being in the context of climate change [21].

**Figure 1:**
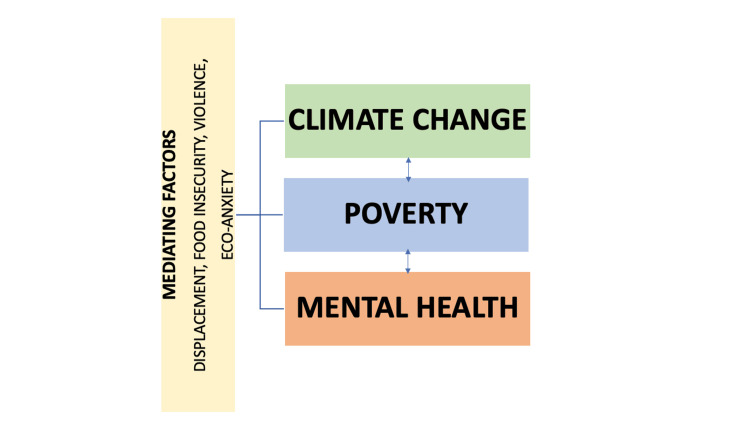
Theoretical framework - Climate change, poverty and mental health Flowchart showing climate change and its impacts. Image Credit: Original illustration created by the authors.

The conceptual framework linking climate change, poverty, and mental health is depicted in Figure 1.

This narrative review aims to summarize existing evidence on how climate change, poverty, and mental health are interconnected, with a view to their interdependence and compounding effects on one another. It seeks to determine vulnerable communities and at-risk populations who face a disproportionate burden of stressors related to climate hazards because they fall into socioeconomic, geographical, and health disparities and explore the mechanisms through which climate change leads to deepened poverty and the potential for adverse mental health outcomes, such as new presentations such as trauma in response to weather events or concerns about local conditions linked to eco-anxiety. This review also aims to assess the implications of current policy frameworks and interventions for climate resilience, poverty alleviation, and mental health as they pertain to inclusivity, gender sensitivity, and cross-sectoral coordination.

## Review

Methodology

Despite the growing recognition of the overlapping domains of climate change, poverty, and mental health, comprehensive reviews synthesizing evidence across these areas remain scarce, underscoring the need for this narrative review. Between May and August 2025, an extensive review of the peer-reviewed literature was performed. The main databases utilised for this search were PubMed, Scopus, Web of Science, and Google Scholar, employing a mix of keywords and MeSH terms such as “climate change,” “global warming,” “poverty,” “mental health,” “eco-anxiety,” “suicide,” “vulnerability,” and “health systems adaptation.” Boolean operators (AND/OR) were used to combine these terms. Additionally, reference lists from pertinent reviews and original articles were manually examined to identify further studies. Inclusion criteria comprised peer-reviewed original research articles, reviews, meta-analyses, and policy papers published between 1990 and 2025 in English, which examined at least one of the three key domains-climate change, poverty, or mental health and discussed their intersections.

A narrative review was selected to compile extensive multidisciplinary evidence connecting climate change, poverty, and mental health. The existing body of research encompasses fields such as epidemiology, sociology, psychiatry, and policy studies, making systematic or meta-analytic methods less suitable because of the diversity in study designs and outcomes. Narrative reviews facilitate the integration of varied evidence, critical evaluation, and contextual interpretation, drawing attention to research gaps, such as the scarcity of data from low- and middle-income countries and the absence of intersectional analyses. This method offers a thorough understanding of the subject, pinpoints key vulnerabilities, and guides future research directions and policy strategies in a changing global landscape.

Mental health and climate change

With climate change, extreme events have become more common, such as natural disasters and food insecurity. The magnitude of this impact is also evident in mental health, so much so that a new term, climate trauma,” has been formed to describe its effects on the psychological and emotional domains [26]. These impacts encompass a spectrum of mental health conditions, ranging from acute stress responses to long-term debilitating conditions.

The rise in climate change leads to more frequent severe weather events, such as floods, hurricanes, and droughts, which consequently result in higher occurrences of depression, anxiety, and post-traumatic stress disorder [27-29]. A recent meta-analysis reported a pooled prevalence of post-traumatic stress disorder (PTSD) and depression of 29.36% and 28.58%, respectively, in the Southeast Asian population affected by adverse weather events [30]. An Indian systematic review of 70 studies demonstrated consistent associations between extreme weather events and elevated risks of depression, anxiety, and post-traumatic stress disorder (PTSD) [31].

It has also been shown to worsen substance use and mood disorders [32,33]. It exacerbates pre-existing mental illnesses, strains health delivery systems, and multiplies the socio-economic hardships of people and communities. This is because of forced migration, loss of income, and loss of home, all of which are known risk factors for precipitating drug abuse [34]. Exposure to traumatic climate events and their association with cognitive decline have also been reported. Individuals exposed to traumatic climate events, such as wildfires, struggle with decision-making, focus, and emotional resilience, undermining day-to-day activities such as parenting and working [35]. Rising temperatures, pollution in the form of exposure to solvents, and chronic exposure to carbon monoxide and nitrogen oxides have all been associated with cognitive decline [36,37].

A growing body of evidence links rising temperatures to an increased suicide risk. A landmark study showed that every 1 °C increase in the monthly average temperature resulted in a 0.7% rise in suicide rates in the United States and a 2.1% rise in Mexico, projecting an additional 9,000-40,000 suicides by 2050 [38]. In India, heat extremes are particularly deadly, with each 1 °C rise above 20 °C associated with an increase in the annual suicide rate amounting to 67 additional suicides annually, largely among agrarian communities [39]. Seasonal and temperature fluctuations are known to influence mood and mental health outcomes, with studies demonstrating higher rates of depression and suicide during periods of elevated temperature and climatic instability [38, 39]. Projections have predicted increases in suicides on a scale comparable to that caused by major economic recessions [40]. Together, these underscore the lethal intersection of climate stress, socioeconomic vulnerability, and mental health, positioning suicide as a sentinel outcome of climate change.

Global warming multiplies the threat of violence at both the community and household levels. Rising resource scarcity, displacement, and economic strain fuel collective violence and conflict, especially in low-income countries with existing inequalities [41]. At the household level, climate stress amplifies gendered vulnerabilities, as evidenced by increased rates of intimate partner violence [42]. Those with pre-existing mental disorders, such as schizophrenia, are at a three-times higher risk of mortality due to impaired thermoregulation from medication, social isolation, and reduced awareness [43].

Geography acts as a multiplier of inequity. Poor people living in climate change hotspots, such as low-lying coastal areas, floodplains, drought-prone regions, informal settlements, or slums, face recurrent environmental trauma and cumulative stress [44,45]. Occupational exposure is highest among outdoor workers. This leads to a reduction in economic productivity. In the event of a disaster, the first responders are usually doctors and paramedics who face both physical and psychological trauma [46].

**Figure 2:**
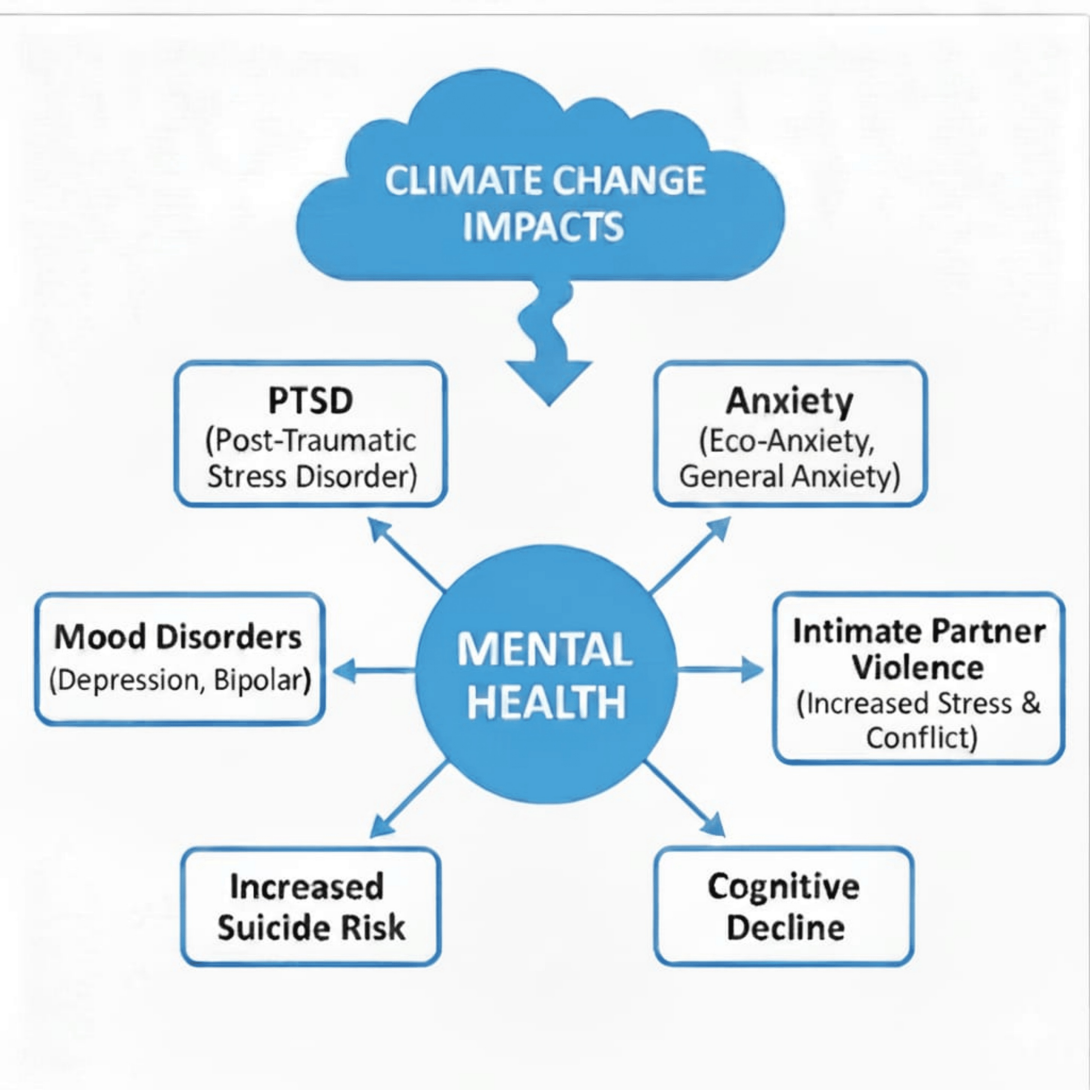
Flowchart showing the impact of climate change on mental health Flowchart showing climate change and its impacts. Image Credit: Original image created by the authors.

Priority for mental health interventions should be given to high-risk populations, such as those with repeated exposure, poor coping mechanisms, and pre-existing illnesses. However, longitudinal studies identifying target groups for long-term mental health interventions are lacking. Policymakers and clinicians should construct early warning systems and undertake community outreach and patient education programs to warn people susceptible to climate change while ensuring that all policies going forward are gender-sensitive and culturally appropriate. Mental health disorders added $2.5 trillion to global health expenses in 2010, a figure that is projected to reach $6 trillion by 2030 [47]. This includes the direct expenses of mental health care services and indirect expenses incurred due to loss of productivity. More studies are required to estimate the precise costs of climate change-induced mental health problems. The overall pathway showing climate change and its impacts is presented in Figure 2.

Although these studies collectively reveal significant links between climatic factors and negative mental health outcomes, much of the evidence comes from cross-sectional or ecological analyses, which limit the ability to draw causal conclusions. Factors such as socioeconomic stress, displacement, and existing mental disorders often overlap with climatic exposure, complicating attribution. Nonetheless, the consistent findings across various populations indicate that seasonal and temperature-related changes have a noticeable impact on mood and behavior.

Poverty and climate change 

Although poor countries contribute the least to climate change, it acts as a poverty multiplier, disproportionately affecting the poorest individuals in these nations [48-50]. Without inclusive economic growth, social security, and healthcare for the most impoverished, climate change is projected to push 132 million people into poverty by 2030. In India, around 700 million people rely on climate-sensitive livelihoods, such as agriculture and fisheries, making them particularly vulnerable to global warming. Furthermore, climate change exacerbates poverty through three main pathways: increasing disease burden, food price inflation, and frequent climate-change-induced disasters such as floods and cyclones [51-53].

Floods, which are responsible for a mortality rate 23 times higher in low-income countries than in high-income countries, exacerbate the shortage of clean drinking water. This shortage, along with overcrowding, forced displacements, and poor sanitation, contributes to outbreaks of diarrheal diseases, Hepatitis A and E, and leptospirosis [54,55]. The precarious food security situation in many parts of the world is worsened by the lack of robust irrigation infrastructure [55]. Droughts lead to widespread crop failures, spiking food prices, and even force smallholder farmers to switch occupations due to reduced income, as seen in Bangladesh. Together, these factors fuel malnutrition as the poorest attempt to reduce expenditure [56]. Poor households often live in poorly ventilated or heat-trapping homes (e.g., tin roofs, slums), where heatwaves expose them to heat exhaustion, stroke, preterm labour, and increased mortality, especially among vulnerable populations [57]. Globally, 80% of the 7.3 billion people exposed to unsafe concentrations of PM2.5 hail from low- and middle-income countries. The poorer population also uses biomass fuels and consequently suffers from respiratory illnesses [58].

Policy responses can protect against these risks. At the individual level, cash transfers, microloans, and subsidized crop and health insurance act as buffers against climate shocks. Programs like the R4 Rural Resilience Initiative act at the individual and community levels to protect the poorest from climate shocks through a four-pronged strategy: community labor-for-assets programs, such as soil conservation programs; weather-based insurance that automatically pays farmers based on satellite weather and vegetation data; livelihood diversification; and financial training and microloans through village savings groups to protect households from asset sales during disasters. Nationally, universal health coverage schemes, such as Ayushman Bharat in India, buffer the poorest people from climate-related health shocks [59,60]. Integrating climate risk into national planning, infrastructure, and health delivery systems, such as through the WHO framework for climate-resilient health systems, ensures continuity of care during natural disasters and improves the adaptive capacity of the poor to climate hazards [61].

India's key strategy for addressing climate change is the National Action Plan on Climate Change (NAPCC), which was launched in 2008. The NAPCC consists of eight missions focusing on solar energy, energy efficiency, sustainable habitats, water resource management, protection of the Himalayan ecosystem, afforestation (Green India), climate-resilient agriculture, and strategic climate knowledge development [62,63]. Currently, India is developing its first National Adaptation Plan (NAP) to enhance resilience among vulnerable communities and integrate adaptation into national planning processes [64]. Supporting schemes, such as the Climate Change Action Programme (CCAP) and the National Adaptation Fund for Climate Change (NAFCC), provide financial and technical aid for climate adaptation at the state level. To further boost climate action, the government launched the Green Credit Program, which promotes voluntary pro-environmental behaviour through a tradable credit mechanism [65]. Additionally, the government approved the establishment of carbon capture and utilisation (CCU) testbeds in the cement industry to demonstrate net-zero emission pathways for hard-to-abate sectors [66,67].

Together, these efforts reflect India's alignment with its commitments under the Paris Agreement and its long-term goal of achieving net-zero emissions by 2070 while fostering sustainable development across sectors. India’s journey toward carbon neutrality faces significant challenges stemming from its expanding population, rising energy demand, and the complex relationship between energy intensity and carbon emissions. Although the adoption of green technologies may temporarily raise emissions, these initiatives are expected to yield substantial long-term benefits by reducing overall carbon intensity. Strengthening energy efficiency is crucial, as it offers medium- and long-term emission reductions, although the impacts may vary across sectors. To overcome these challenges, India must broaden its low-carbon energy portfolio, accelerate the deployment of clean technologies, and enhance public awareness through behavioural change initiatives such as LiFE (Lifestyle for Environment). The nation’s success in achieving its 2070 net-zero target will depend on maintaining the balance between economic growth, social development, and environmental sustainability [68].

India's journey towards achieving carbon neutrality by 2070 faces hurdles such as its expanding population, temporary rises in emissions due to the adoption of green technologies, and the intricate link between energy intensity and carbon emissions. To address these challenges, India must broaden its low-carbon energy initiatives, increase the deployment of green technologies, and establish effective public awareness campaigns. The nation's success will hinge on its ability to balance these elements while sustaining economic growth and social progress. The NAPCC offers a comprehensive framework that addresses both mitigation and adaptation strategies through its eight core missions, which focus on renewable energy, water, agriculture, urban sustainability, and ecosystem conservation [69]. It embodies India’s commitment to sustainable development, integrates climate concerns into national policy, and aligns with global frameworks such as the Paris Agreement [70]. The emphasis on decentralized implementation through state action plans ensures contextual relevance [71]. Despite its vision, the NAPCC faces challenges such as a lack of inter-ministerial coordination, limited budgetary allocation, and inadequate monitoring and evaluation mechanisms [70]. Missions such as the National Mission on Sustainable Habitat and the Green India Mission have reported significant implementation delays and poor fund utilisation [72]. Additionally, there is insufficient attention to social vulnerability, gender impacts, and community engagement, which reduces the inclusivity of the plan [70]. With the rise in global climate finance and India’s evolving green economy, the NAPCC has the potential to integrate climate resilience into infrastructure development, promote green jobs, and attract investments in clean energy and carbon capture and utilisation (CCU) technologies [73]. The formulation of India’s first National Adaptation Plan presents an opportunity to bridge adaptation gaps and enhance the integration of local governance [69]. The growing climate risks, particularly extreme weather, glacial retreat, and drought, pose urgent implementation demands. Challenges include data inadequacy, technical capacity constraints, and the need for sectoral convergence at all administrative levels [70]. Moreover, aligning the NAPCC with newer initiatives, such as the Green Credit Programme and State Action Plans on Climate Change (SAPCCs), necessitates dynamic policy evolution and robust institutional support [73].

Although numerous adaptive and social protection strategies have been put into place, their success rates differ significantly. Initiatives such as the R4 Rural Resilience Initiative and Ayushman Bharat show encouraging results in mitigating risks and safeguarding health, yet their reach is inconsistent, and financial sustainability poses a major challenge to expansion. Likewise, projects under India’s National Action Plan on Climate Change (NAPCC) encounter obstacles related to coordination among ministries, community involvement, and the efficient use of funds. There is a scarcity of data on the actual impact of these programs, emphasizing the necessity for structured monitoring and evaluation systems. In contrast, wealthier nations have managed to establish more forward-thinking climate adaptation and insurance-based strategies due to their robust institutional frameworks and larger financial resources. This difference highlights an increasing adaptation gap between affluent and less affluent countries, exacerbating global disparities in resilience. Bridging these gaps in implementation and evaluation is vital to ensure that climate and poverty alleviation policies lead to real health and economic improvements in low- and middle-income settings.

Limitations

This review has certain limitations. As a narrative review, it did not use systematic selection or quality assessment criteria, which could lead to selection bias. Most of the evidence available is cross-sectional, making it challenging to determine causality between climate change, poverty, and mental health outcomes. Additionally, the literature is heavily skewed towards high-income countries, with limited data from low- and middle-income areas where vulnerabilities are most pronounced. The diversity in study designs and outcomes, along with the absence of gender-disaggregated and intersectional analyses, limits comparability and generalizability. The focus on short-term, event-based studies also hinders the understanding of long-term psychosocial adaptation and resilience. These methodological limitations may have led to interpretation bias, as the conclusions are based on studies of varying quality.

Future research should employ longitudinal and mixed-method approaches to capture evolving community responses and intergenerational impacts. Incorporating economic modelling, spatial analysis, and participatory frameworks would enhance causal inference and policy relevance. Examining diverse theoretical perspectives, such as the social-ecological model, syndemic theory, and intersectionality, can further clarify how climate stress interacts with social inequities and health outcomes. Engaging with conflicting evidence, including studies on resilience and adaptive coping, will provide a more balanced understanding and guide intervention design. Policy and practice must progress toward equity-centred strategies that address the interconnected risks of climate stress, poverty, and mental illness. Expanding community-based interventions, such as livelihood diversification, cash transfers, and climate-resilient health services, is crucial. Integrating mental health within climate adaptation and mitigation policies, improving early warning systems, and training frontline workers in culturally sensitive care are key priorities. Strengthened intersectoral collaboration across the health, environment, and social protection sectors, and gender-responsive policies, is essential to ensure inclusive and sustainable resilience.

## Conclusions

Climate change amplifies poverty and mental health challenges, particularly among vulnerable populations. Ultimately, dismantling the reinforcing cycle of climate change, poverty, and mental health challenges demands a multi-level, integrated response rooted in equity. At the policy level, this necessitates immediate, system-wide action: integrating mental health services into all climate adaptation and disaster risk reduction plans, legislating gender-responsive policies to address disproportionate burdens, and strengthening universal health coverage schemes, such as Ayushman Bharat, to buffer the poorest from climate-related health shocks. At the community level, emphasis must be placed on scaling up resilience initiatives such as the R4 Rural Resilience framework, which combines microloans and climate-based insurance, alongside comprehensive community outreach and patient education programs. Crucially, the individual level is empowered through education on climate awareness and mental health management tools. Through these changes, we can foster the holistic resilience required to protect the most vulnerable populations in an increasingly complex and warming world.
